# A follow-up study of vibration-induced injuries in workers exposed to transient and high frequency vibrations

**DOI:** 10.1186/s12995-024-00425-6

**Published:** 2024-06-21

**Authors:** Lars Gerhardsson

**Affiliations:** Department of Occupational and Environmental Medicine, School of Public Health and Community Medicine, Institute of Medicine, Sahlgrenska Academy, University of Gothenburg, Medicinaregatan 16 A, Box 414, Gothenburg, SE-405 30 Sweden

**Keywords:** Vibration white fingers, Neurosensory disturbance, Musculoskeletal symptoms, High frequency vibrations

## Abstract

**Background:**

In a previous study from 2018, 38 wheel loader assembly workers were examined, showing high exposures to transient and high-frequency vibrations. After the investigation, preventive measures were immediately implemented to reduce the vibration exposure. In 2022, a follow-up study was carried out to examine the effect of these measures.

**Methods:**

The follow-up study included 35 (27 men and 8 women) of the original 38 workers. They were divided into two groups, 24 workers with ongoing vibration exposure and 11 workers, not vibration exposed since 2018. All participants completed a questionnaire and underwent a thorough examination, including several neurophysiological tests and a comprehensive assessment of musculoskeletal symptoms. The questionnaire responses and on-site vibration level measurements formed the basis for the individual vibration exposure assessment.

**Results:**

In 2018, clear differences were noted between the two groups regarding vibration perception thresholds (VPT), needle test, 2-PD (2-point discrimination), and monofilament test with deviating results in the unexposed group. The difference between the two groups was significantly smaller at the follow-up examination in 2022, where differences remained for VPT and monofilament tests, with deviating test results in the unexposed group. When comparing variable values between 2018 and 2022 within the exposed and unexposed groups, respectively, the unexposed group showed mostly unchanged values, while a deterioration was observed for VPT, needle test and temperature sensitivity test among the exposed workers during follow-up. The prevalence of VWF (Vibration white fingers) was around 30–40% and neuropathy around 75% among exposed workers during follow-up compared to about 60% and 85% respectively, in the unexposed group.

**Conclusion:**

The overall categorization of white fingers and neuropathy, according to the Stockholm Workshop Scale, remained largely unchanged in both study groups from 2018 to 2022. The introduction of cost-effective and relatively simple preventive measures may have contributed to this result. Throughout the follow-up period, the number of exposed workers who developed musculoskeletal disorders and newly reported cases of vibration injuries at the factory decreased. Without this preventive program, increased vascular and nerve symptoms would most likely have occurred during follow-up due to continued vibration exposure.

## Introduction

Vibration exposure can cause the hand-arm vibration syndrome (HAVS), which is characterized by vibration white fingers (VWF), tingling and numbness in fingers and hands, reduced grip strength and decreased manual dexterity [1]. Factors such as the duration and intensity of exposure can increase the risk to develop this condition [2].

A high prevalence of HAVS has been observed at some workplaces despite a low daily vibration exposure. In the aircraft industry a prevalence of vibration white fingers (VWF) of approximately 50% was found among riveters with more than ten years of vibration exposure [3]. In a study of Swedish truck mechanics, mainly using impact wrenches, the prevalence of VWF was about 25% and the prevalence of neurosensory disturbances about 40% after 20 years of exposure [4]. The daily vibration exposure time in both studies was low but the vibrations from the tools were characterized by impacts and shock wave accelerations.

Neurosensory disturbances have a worse prognosis than vascular changes. They usually appear earlier and last longer than the vascular changes. At equal exposures, neurosensory disturbances occur with a three-time factor shorter latency compared to the vascular changes [5].

The duration of hand-arm vibration symptoms is dependent on the individual sensitivity of the exposed workers and the grading of the symptoms. Some workers can develop quite severe symptoms after just a few years of exposure, while other workers can be exposed for decades without other than minor symptoms. Also, the grading of the vascular and neurological symptoms is of importance. A high grade means severe symptoms and a longer time to recover if the vibration exposure is ceased. In a Danish study, finger systolic blood pressure improved in nearly half of the studied population after an observation period of one to 13 years. The highest improvement was found in subjects with little or no exposure during the last two years, in non-smokers and in subjects with no other circulatory diseases than VWF [6]. Work with hand-held tools with high frequency vibrations, smoking, other circulatory diseases, and low age at the time of diagnosis affected the prognosis in an unfavourable way [6].

Several studies have reported a high level of upper extremity disorders in vibration exposed workers [7–10]. The pathogenesis of these disorders in users of vibrating tools is not yet fully clarified. When working with vibrating tools workers are exposed to risk factors for musculoskeletal disorders such as grip and push forces, repetitive motions, non-neutral postures, and manual handling. Age, hard physical workload and poor psychosocial well-being increased the risk of developing musculoskeletal disorders in neck and upper limb. With so many contributing factors, all parameters of the work system must be considered, e.g. tasks, environment, organisation, and technology, when studying musculoskeletal symptoms among workers exposed to hand-arm vibration [9]. It is also known that the muscle activity is increased by vibrations. This phenomenon is called tonic vibration reflex (TVR) [11].

In 2018, we studied a population of wheel loader assembly workers exposed to transient and high-frequency hand-arm vibrations [12]. We found a high prevalence of VWF and neurosensory findings. Furthermore, musculoskeletal findings were common e.g., carpal tunnel syndrome and ulnar entrapment in hand/wrist, especially in male workers.

This study is part of a larger project named Zero vibration injuries, with the aim to reduce the vibration levels of handheld machines. Shortly after the study in 2018, the factory started to modify the tools used at the assembly line to substantially reduce the workers’ high frequency vibration exposure. The present investigation is a 4-year follow-up study of the same population of wheel loader assembly workers that we studied in 2018, to evaluate the effect of these preventive measures.

## Methods

### Aims of the study

The goal of this four-year follow-up study was to assess whether a program designed to decrease the exposure to transient and high frequency hand-arm vibrations could impact the occurrence of HAVS and musculoskeletal symptoms among assembly workers.

### Study population

At baseline in 2018 the study population consisted of 38 assembly line workers (30 men, 8 women). At the follow-up in 2022, three workers, all men, were not available due to long-term sickness, retirement, and job change, which meant that the population in this study consisted of 35 workers (27 males and 8 females). The mean-age among the males was 43 ± 9 yrs. compared to 43 ± 11 yrs. among the females and the mean exposure time was 17 ± 7 yrs. and 18 ± 8 yrs. respectively.

We monitored two groups over a four-year follow-up period starting in 2018. Twenty-four workers continued their daily tasks until 2022, while 11 workers at the beginning of the follow-up had such severe symptoms of white fingers and neurosensory symptoms that they had to be reassigned to non-vibration-exposed tasks. In the unexposed group most subjects worked up to 2018, but not after that. A few workers in this group had stopped working a year or two before 2018. This allowed us to follow and compare the two groups from 2018 to the study’s conclusion in 2022.

### Study design

At baseline in 2018, the workers completed a questionnaire about work, medical history, exposure time, type of vibrating tools and hand-arm vibration symptoms.

A medical examination was performed by an experienced physician including 2-PD test (2-point discrimination), Semmes-Weinstein’s monofilament test (5 filament kit), sensitivity to pain (needle test) and test of hand grip strength (Jamar). Neurosensory tests also included the determination of vibration perception thresholds (VPTs) and the determination of the sensitivity to cold and warmth by the Rolltemp© II instrument [13]. The symptoms and signs of vascular and neurosensory disturbances were graded according to the Stockholm Workshop Scale (SWS). The vascular scale is graded from 0 to 4. The neurosensory scale goes from 0 SN to 3 SN. The higher grade, the more serious is the disease. The participants were told to avoid vibration exposure during the day of the measurement as well as intake of tobacco and coffee at least one hour before the medical tests. These test methods are described in detail in Gerhardsson et al. 2020 [12]. At the follow-up in 2022, the workers answered the same questionnaire as in 2018 and underwent the same medical examination. Also, the medical investigators were the same as in 2018.

During the VPT testing, the participants used ear protection devices to exclude noise from indoor and outdoor sources. A sensibility index (SI-index) was calculated by dividing the area under the curve down to 150 dB for the participant with the corresponding area for the reference population. An SI-index < 0.8 is interpreted as an abnormal response. An excellent reliability (ICC > 0.94) [14] has been observed in VPT-determinations in patients with diabetic neuropathy.

### Cut off limits

To interpret the test results, the cut-off limits used are presented in Table 1.

**Table 1:** Cut-off limits for the medical tests

Medical test	Normal	Deviant
2-PD (mm)	≤ 5 mm	> 5 mm
Monofilament (0.07–300 g)	≤ 0.2 g	> 0.2 g
Temperature sensitivity	0	1
Needle test (sharp/blunt)	0	1
Jamar (kg)	Male RH ≥ 45, 42, 32 kg Male LH ≥ 40, 39, 26 kg	(< 40, 55, 65 years) (< 40, 55, 65 years)
Sensibility index VPT	≥ 0.8	< 0.8
Stockholm Workshop Scale		
Vascular grade (0–4) Neurosensory grade (0 SN – 3 SN)	0-1 0-1 SN	2-4 2-3 SN

### Musculoskeletal examination

The same musculoskeletal examination of the neck, shoulders, arms, and hands was performed in 2018 and 2022 according to the HECO-protocol (Health Surveillance in Adverse Ergonomics Conditions, MEBA in Swedish) by an experienced physiotherapist as earlier described by Gerhardsson et al. (2020) and Jonker et al. (2015) [12, 15]. 

### Work ability index

Work ability index (WAI) is a concept that was developed in Finland during the 1980’s [16]. We have used the first item from the WAI questionnaire, named work ability score (WAS). It is a self-assessment of current work ability compared to lifetime best. It has a strong correlation and an equally good predictive value regarding, e.g. sick leave, health and reported pain as the whole WAI-instrument [17, 18].

### Ethical approval

The study was approved by the Swedish Ethical Review Authority at the city of Uppsala, Sweden. All participants signed a written consent and completed a basic questionnaire with questions about e.g. work and medical history, exposure time, type of vibrating tools and hand-arm vibration symptoms. The questions were focusing on the four-year follow-up period.

### Vibration exposure

#### Tool modifications

Modified tools that substantially reduced the workers’ high frequency vibration exposure were introduced at the assembly line shortly after the baseline study in 2018. The original impact wrench handles were covered with a three millimetres thick foamed polymer with closed cells on the handle. The redesigned counter torque spanners had a vibration isolating layer consisting of a foamed polymer with a thickness of about five millimetres between the part holding the nut and the handle [19]. The redesigned tools are ½ inch impact wrenches of oil pulse type (Atlas Copco Ergo pulse) and counter torque spanners in form of ordinary wrenches of sizes 10–13 mm. The vibration exposure according to ISO 5349-1 was calculated to track potential changes of hand arm-weighted vibration exposures between baseline and follow-up. The vibration reduction activities are mainly focused on reducing high acceleration peaks with a high frequency content.

#### Vibration levels

The tool vibration for the ISO 5349-1 weighted rms was the same before and after the redesign of the impact wrench (4 m/s^2^_hav_), but the high frequency vibration amplitude was reduced to about 20% of the original (from 2000 to 400 peak m/s^2^). The re-design of the counter torque spanner reduced the hand-arm weighted vibration level to about half and the high frequency content to less than a 30th of the original [19]. Hand-arm weighted vibration levels for other tools than impact wrenches and counter torque spanners were taken from the assembly plant inventory lists.

At baseline, the exposure time was self-reported, and at follow-up, the exposure time was calculated based on the estimated number of nuts and bolts in production and on-site observations. A(8) at baseline was 0.6/0.9 m/s² right/left hand if evaluated in the same way as at follow-up (0.5/0.6 m/s² right/left hand).

The ISO vibrations decreased for the counter torque spanner, but not for the impact wrench. Other differences may be due to participants changing workstations or tempo between baseline and follow-up.

#### Assessment of vibration exposure

##### Exposure times

The use of the modified tools varied among the exposed workers. Some of the workers mostly used the old tools, while others used the modified tools most of their working time.

The vibration exposure time at baseline and follow-up was calculated from components lists of the average vehicle produced at the assembly line. The component lists contained detailed information of the number of screws, screw types and nuts used at each workstation along the assembly line and could be used to calculate the exposure time for impact wrenches, counter torque spanners and screwdrivers. Observations were made at the assembly line workstations to verify the list-based method. The average time to tighten a screw or nut was derived from the measurements in 2019 [19] and from observations at the assembly line. Approximately 150 nuts were tightened by the worker at each shift, and thus the whole exposure time is just a few minutes. Measurements show that the high frequency vibration amplitude of the modified tools was considerably reduced [19].

However, there is no established measurement and evaluation method for assessment of high frequency vibration exposure.

### Statistics

Normal probability plots and Levene’s test were used to test the normality of the input variables. Because most of the variables showed a skewed distribution, non-parametric statistics were chosen for the statistical calculations.

Mann-Whitney U-test was used to test for differences between two independent groups on a continuous measure.

Wilcoxon’s Matched Pairs Signed Ranks Test was used to compare repeated measures, which is values from the same worker, measured with the same scale at two occasions, 2018 and 2022, respectively.

Group differences between exposed (*N* = 24) and unexposed (*N* = 11) workers for single variables measured in 2018 and 2022 was evaluated with Fisher’s exact test. *P*-values < 0.05 were regarded as statistically significant.

A multiple regression analysis was performed to evaluate which individual and work-related factors that showed the strongest connection with work ability score (WAS).

All calculations were performed with IBM/SPSS statistics 28.0 [20].

The chi-square test for independence has been used for the testing of 2-PD, monofilament, temperature sensitivity test, needle test and SWS tests. The significance of the results was expressed as Fisher’s exact test instead of expressing the chi-square value, as one or more of the cell counts in a 2 × 2 table was less than 5.

For the comparison of Jamar and sensibility index (vibration perception thresholds) tests the Mann-Whitney U-test was used.

In Table 2, data from two dichotomous variables (0 vs. 1) was presented, temperature sensitivity test and needle tests. For these variables 0 denotes a normal response and 1 a deviant response. Accordingly, mean values close to 0 shows higher normality than mean values close to 1.

**Table 2:** Results from the neurosensory testing of vibration exposed and unexposed workers

Variables	2018	2022	*N* = 11	2018	2022	*N* = 24
	Mean ± SD	Mean ± SD	*p*-values	Mean ± SD	Mean ± SD	*p*-values
2PD dig 2 R	4.6 ± 1.0	4.9 ± 1.1	NS	4.1 ± 0.3	4.5 ± 0.9	NS
2PD dig 5 R	5.3 ± 1.5	5.3 ± 1.0	NS	4.1 ± 0.6	5.1 ± 1.1	NS
2PD dig 2 L	4.6 ± 1.0	4.7 ± 0.9	NS	4.0 ± 0.0	4.3 ± 0.7	NS
2PD dig 5 L	4.8 ± 1.1	5.1 ± 1.2	NS	4.0 ± 0.2	4.8 ± 0.8	NS
Mono dig 2 R	3.6 ± 0.6	3.5 ± 0.7	NS	3.3 ± 0.4	3.2 ± 0.5	NS
Mono dig 5 R	3.8 ± 1.1	3.7 ± 1.2	NS	3.1 ± 0.4	3.1 ± 0.5	NS
Mono dig 2 L	3.7 ± 1.1	3.5 ± 0.7	NS	3.3 ± 0.5	3.2 ± 0.5	NS
Mono dig 5 L	3.6 ± 0.5	3.5 ± 1.2	NS	3.2 ± 0.4	3.1 ± 0.5	NS
Temp dig 2 Rw	0.3 ± 0.5	0.4 ± 0.5	NS	0.2 ± 0.4	0.5 ± 0.5	0.021
Temp dig 2 Rc	0.4 ± 0.5	0.6 ± 0.5	NS	0.1 ± 0.4	0.6 ± 0.5	< 0.001
Temp dig 5 RwW	0.5 ± 0.5	0.4 ± 0.5	NS	0.3 ± 0.5	0.5 ± 0.5	NS
Temp dig 5 RcC	0.4 ± 0.5	0.6 ± 0.5	NS	0.3 ± 0.5	0.7 ± 0.5	0.039
Temp dig 2 LwW	0.2 ± 0.4	0.4 ± 0.5	NS	0.1 ± 0.3	0.5 ± 0.5	0.002
Temp dig 2 LcC	0.1 ± 0.3	0.6 ± 0.5	0,031	0.1 ± 0.3	0.5 ± 0.5	0.039
Temp dig 5 LwW	0.3 ± 0.5	0.5 ± 0.5	NS	0.1 ± 0.3	0.5 ± 0.5	0.021
Temp dig 5 Lc-C	0.2 ± 0.4	0.6 ± 0.5	NS	0.2 ± 0.4	0.6 ± 0.5	0.003
Vibb dig 2 R	0.75 ± 0.43	0.73 ± 0.28	NS	1.09 ± 0.16	0.91 ± 0.16	< 0.001
Vibb dig 5 R	0.66 ± 0.43	0.68 ± 0.30	NS	1.04 ± 0.18	0.84 ± 0.18	< 0.001
Vibb dig 2 L	0.74 ± 0.40	0.77 ± 0.38	NS	1.12 ± 0.17	0.96 ± 0.15	< 0.001
Vibb dig 5 L	0.70 ± 0.40	0.62 ± 0.37	NS	1.06 ± 0.17	0.86 ± 0.19	< 0.001
Jamar LH	36 ± 9	38 ± 13	NS	46 ± 14	46 ± 14	NS
Jamar RH	38 ± 8	38 ± 10	NS	47 ± 15	48 ± 13	NS
Needle dig 2 R	0.5 ± 0.5	0.5 ± 0.5	NS	0.04 ± 0.20	0.6 ± 0.5	< 0.001
Needle dig 5 R	0.4 ± 0.5	0.5 ± 0.5	NS	0.08 ± 0.28	0.3 ± 0.5	0.031
Needle dig 2 L	0.5 ± 0.5	0.5 ± 0.5	NS	0.04 ± 0.20	0.6 ± 0.5	< 0.001
Needle dig 5 L	0.4 ± 0.5	0.5 ± 0.5	NS	0.04 ± 0.20	0.4 ± 0.5	0.008
SWS VWF R	1.00 ± 1.00	1.27 ± 1.27	NS	0.42 ± 0.78	0.38 ± 0.71	NS
SWS VWF L	1.00 ± 1.00	1.27 ± 1.27	NS	0.42 ± 0.78	0.54 ± 0.83	NS
SWS N-sens R	1.64 ± 1.03	1.45 ± 1.29	NS	0.88 ± 0.74	1.00 ± 0.89	NS
SWS N-sens L	1.64 ± 1.03	1.45 ± 1.29	NS	0.79 ± 0.78	0.96 ± 0.91	NS

Table 2. Values in vibration exposed workers (*N* = 24) and in unexposed workers (*N* = 11) in 2018 and 2022. *P*-values from repeated measurements of values 2018 vs. 2022 within the same group. The following variables were determined: 2-PD – 2 point discrimination in mm; Mono = Semmes Weinstein’s monofilament test (0.07 g, 0.2 g, 2 g, 4 g, 300 g); Temp = Temperature sensitivity measured by Rolltemp II

(0 = normal; 1 = deviant response), Vib –vibration perception thresholds (VPT) expressed as Sensibility Index (SI < 0.8 is deviant;  SI ≥ 0.8 is normal.), Needle = needle tests, is a sharp needle experienced as sharp or blunt (0 = sharp; 1 blunt, deviant); Classification of VWF as 0 to 4 and classification of neuropathy as 0 SN to 3 SN according to the Stockholm Workshop Scale. Jamar (kg). R = right hand: L = left hand. VWF = vibration white finger. c = cold, w = warmth

## Results

The material consists of two original groups, a vibration-exposed group (*N* = 24), and an unexposed group (*N* = 11; Table 2). The exposed and unexposed groups were compared with each other both at the baseline examination in 2018 and at the follow-up examination in 2022. Additionally, the variable values in 2018 were compared with the corresponding values in 2022 within the same group (repeated measures).

Significant differences occurred when all exposed workers were compared with the unexposed group. In 2018, significantly lower SI-indices were observed over the index and little finger bulbs bilaterally in the unexposed group (dig 2 dx; *p* = 0.018; Mann-Whitney; dig 5 dx; *p* = 0.021; M-W; dig 2 sin; *p* = 0.005; M-W and dig 5 sin; *p* = 0.009; M-W) compared to all exposed workers. Differences and more deviating values among the unexposed group were also noted for the needle tests (blunt/sharp) in dig 2 dx (*p* = 0.007; Fisher’s exact test) and in dig 2 (*p* = 0.007; Fisher) and dig 5 (*p* = 0.026; Fisher) in the left hand. Furthermore, significantly more deviating values were found for 2-PD in dig 5 dx (*p* = 0.007; Fisher) and dig 5 sin (*p* = 0.025; Fisher) as well as for Semmes Weinsteins’s monofilament test in dig 5 dx (*p* = 0.025; Fisher) in the unexposed group compared to exposed workers. No differences between the groups were noted for the other variables.

The differences had decreased when the same groups were compared at the follow-up examination in 2022. Differences were noted for VPTs in dig 2 dx (*p* = 0.033; M-W) with the lower value in the unexposed group. Likewise, higher and more deviating values were noted for monofilament tests in dig 2 dx (*p* = 0.026; Fisher) and dig 5 dx (*p* = 0.026; Fisher) in unexposed workers. No significant differences between the groups were observed for other variables.

Variable outcomes in 2018 were also compared with corresponding variable outcomes in 2022 in the unexposed group (repeated measures; Table 2). As shown in the table, no significant differences were observed between 2018 and 2022 for any of the variables in the unexposed group except for a deterioration of temperature sensitivity for cold in dig 2 left hand (*p* = 0.031).

A different outcome was seen in all exposed workers (Table 2). No significant differences were seen for 2-PD tests, monofilament tests, hand grip strength and for the development of VWF and neurosensory disturbances in both hands according to the Stockholm Workshop Scale. Clear deteriorations in 7 of 8 variable values were noted for temperature sensitivity (*p* ≤ 0.039), and for all four VPTs and all four needle tests during the follow-up period (Table 2).

The prevalence of vibration white fingers (VWF) in unexposed workers varied from 64% in 2018 to 55% in 2022. Corresponding prevalence figures for neurosensory disturbances varied from 91% in 2018 to 82% in 2022. The prevalences of VWF in exposed workers varied from 29% in 2018 to 36% in 2022 and the prevalence of neurosensory disturbances varied from 75% in 2018 to 76% in 2022. However, none of the changes showed a significant result.

The grading of vibration white fingers and neuropathy in the studied groups are shown in Table 3. As can be seen from the table, the number of subjects in the corresponding cells is roughly the same when comparing left and right hands among exposed and unexposed workers, in 2018 and 2022, respectively.

**Table 3:** Vascular and neurosensory classification in exposed and unexposed workers according to the Stockholm Workshop Scale

SWS	2018				2022			
	*N* = 24 RH	*N* = 24 LH	*N* = 11 RH	*N* = 11 LH	*N* = 24 RH	*N* = 24 LH	*N* = 11 RH	*N* = 11 LH
SWS 0	18	18	5	5	18	16	5	5
SWS 1	2	2	1	1	3	3		1
SWS 2	4	4	5	5	3	5	4	5
SWS 3							2	
SWS 0 SN	7	9	1	1	7	8	3	1
SWS 1 SN	14	12	5	5	12	11	4	5
SWS 2 SN	2	2	2	2	3	3		2
SWS 3 SN	1	1	3	3	2	2	4	3

Table 3. Grading of vibration white fingers and neurosensory findings in 2018 and 2022 according to the Stockholm Workshop Scale. SWS = Stockholm Workshop Scale for vascular changes. SWS SN = Stockholm Workshop Scale for neurosensory changes

RH = Right hand. LH = Left hand. The numbers show the number of subjects in each cell

As expected, most symptoms are observed in the unexposed group.

The vibration-induced changes described above may affect work ability. The exposed workers had a WAS-value of 8.3 ± 1.4 in 2018. It increased to 8.5 ± 1.4 in 2022. In the unexposed group a larger increase was noted from 6.7 ± 2.3 to 7.9 ± 1.7 during follow-up, however not significant. Thus, the working capacity was quite high in both groups of workers during the whole follow-up period.

Previous studies have shown that reduced grip strength has a considerable impact on WAS. Another important factor in this context is age as WAS usually decreases with age. The impact of these two factors were tested by multiple regression analyses. The final model in the right hand had the following equation:

WAS = 11.2–0.05 x age − 0.7 x feeling of reduced grip strength (R^2^ = 0.49; *p* < 0.001). Feeling of reduced grip strength (Beta − 0.62) gave the strongest unique contribution to explaining the dependent variable when the variance explained by all other variables in the model was controlled for. The Beta value for age (-0.31) was lower, indicating that it made less of a unique contribution. Similar values were noted in the left hand. Other tested variables such as e.g. pain in hands and fingers, cramps in arms and hands and vibration exposure time were not included in the model.

### Musculoskeletal findings

In the unexposed group the prevalence of reported musculoskeletal symptoms during the last seven days in the neck-shoulder area decreased with approximately 40%, and in hand/arms with about 50% during follow-up (Table 4). The prevalence of neck-shoulder and hand/arm symptoms during the last 7 days decreased with about 15–20% during follow-up among vibration exposed workers. The largest reduction was observed for the diagnoses tension neck syndrome and biceps tendinitis. The percentage of neck-shoulder diagnoses decreased from 33% in 2018 to 13% in 2022 among vibration exposed workers but was unchanged (9%) in the unexposed group. The percentage of hand-arm diagnoses was unchanged in the exposed group during follow-up (17%) but decreased from 27 to 18% in the unexposed group.

**Table 4:** Musculoskeletal symptoms in neck-shoulder and hand-arms in vibration exposed and unexposed workers

	Exposed group *n* = 24		Unexposed group *n* = 11	
	Baseline	Follow-up	Baseline	Follow-up
Neck-shoulder				
Prevalence of symptoms last 7 days (%)	71	58	45	27
Workers with at least 1 diagnosis (%)	33	13	9	9
Diagnosis (nb)*				
Tension neck syndrome	5	1	1	
Thoracic outlet syndrome	2			
Acromioclavicular syndrome	1	1		1
Biceps tendinitis	4	1		
Supraspinatus tendinitis	3	1		
Infraspinatus tendinitis	3			
Hand-arm				
Prevalence of symptoms last 7 days (%)	63	54	73	36
Workers with at least 1 diagnosis (%)	17	17	27	18
Diagnosis (nb)*				
Lateral epicondylitis			1	
De Quervain’s tendinitis		1		
Overused hand syndrome		1	1	
Radial tunnel syndrome	2			
Ulnar entrapment in elbow	1	2	1	1
Carpal tunnel syndrome	2	4	3	2
Ulnar entrapment in hand/wrist	2	1	2	1

* One worker can have more than one diagnosis

Table 4. Musculoskeletal symptoms and diagnoses in the neck-shoulder and hand-arm regions in the unexposed and exposed groups at baseline and at the 4-year follow-up. The numbers for the presented diagnoses represent the number of subjects in each cell

## Discussion

Comparison of values in 2018 with corresponding values in 2022 in the exposed group showed no differences for 2-PD, monofilament tests, Jamar, and the SWS classification (Table 2).

However, a deterioration was observed for VPTs in the exposed group, which is not surprising as the exposure continued for an additional four years. Deteriorations during follow-up were also seen for needle tests and temperature sensitivity test results.

Several studies have reported increased prevalences of white fingers and neurosensory disorders in workers exposed to transient and high-frequency vibrations [3, 4, 12].

At the baseline study including 38 wheel loader assembly workers, we found high prevalences of white fingers (30% in men; 50% in women) and neurosensory disorders (70% in men; 88% in women). A significant number of musculoskeletal diagnoses (*N* = 32) were also observed among male workers, while the number of diagnoses (*N* = 4) was considerably lower in female workers.

In the 2022 follow-up examination, 35 of the original 38 workers could be studied with the same type of examination as in 2018. Of these wheel loader assembly workers, 24 had been active and vibration-exposed throughout the follow-up period, while 11 workers hade been reassigned to non-vibration-exposed tasks, due mainly to increased complaints of white fingers and neuropathy. As a rule of thumb, classification according to the Stockholm Workshop Scale at stage 2 vascular and stage 2 SN neurosensory implies a recommendation for reduced or discontinued vibration exposure. Stage 3 or 3 SN indicates that the workers should discontinue vibration exposure as soon as possible and change to non-vibration-exposed tasks.

The exposed group (*N* = 24) had a 29% prevalence of white fingers in 2018, which slightly increased to 36% in 2022. However, the prevalence of neuropathy remained stable during the same period around 75%. In the unexposed group (*N* = 11), the prevalence of white fingers was 64% in 2018 and decreased to 55% in 2022. Corresponding prevalence figures for neuropathy were 91% and 82%, respectively. However, even if these prevalences were of the same magnitude as in previous studies [3, 4, 12], no significant differences appeared during follow-up except for the prevalence of VWF in 2018, which showed a marginally significant difference between unexposed and exposed workers.

The unexpectedly high prevalence figures observed during the investigation in 2018 for vibration white fingers and neuropathy started an intense program for workplace improvements at the factory. The key action was focused on the impact wrenches and the counter torque spanners. As earlier described, these measures resulted in a significant reduction in high frequency vibration levels compared to the measured levels in 2018.

Significant differences were found when all exposed workers (*N* = 24) were compared with the unexposed group (*N* = 11) regarding vibration perception thresholds, needle tests, 2-PD and monofilament tests in 2018 and regarding vibration perception thresholds and monofilament tests in 2022. There is, however, a clear difference in sensitivity when comparing different examination methods. Among the tests, VPTs have the highest sensitivity followed by monofilament tests. Needle tests and temperature sensitivity tests have a lower sensitivity and 2-PD has the lowest sensitivity in this context [21].

When the variable outcome in 2018 was compared with the variable outcome in 2022 in the same group, no significant differences were noted in unexposed and exposed groups for 2-PD measurements, Semmes Weinstein’s monofilament test, grip strength, and classification of vascular and nerve changes according to the Stockholm Workshop Scale.

The duration of neurosensory disturbances depends on the individual’s sensitivity and the duration of vibration exposure. In some cases, the duration of neurosensory symptoms can be quite long. Aarhus et al. [22] followed 27 workers with HAVS from 1994 to 2017. They found no statistically significant differences in hand numbness (SWS), shoulder/arm pain (pain scale), finger pain or grip strength from 1994 to 2017. However, they noticed that vibration exposure during follow-up was associated with increased finger pain.

Four years of follow-up is not a long time. Usually, a longer period is needed to see more dramatic positive changes regarding VWF and neurosensory disturbances in the unexposed group. Due to the preventive measures undertaken we got a considerable reduction of vibration exposure in the exposed group, especially for high frequency vibration. However, a low ongoing exposure may still have an effect on these diagnoses, especially for workers with high sensitivity to vibration exposure. Thus, no vibration exposures are absolutely safe for users.

In this case, however, the grading according to the Stockholm Workshop Scale showed an essentially unchanged picture as regards vascular and neurosensory symptoms. No significant deterioration could be detected.

Quantitative sensory testing (QST) is a valuable tool for diagnosing thermal perception thresholds. Vibration exposed subjects showed a lower sensitivity to cold and warmth compared to non-vibration exposed subjects [23]. When using QST, results within the range of two standard deviations from a normative value are usually considered to be normal. The interpretation is, however, not straightforward. Normative threshold values are often obtained from investigations of healthy and non-exposed groups, e.g. students or white-collar workers. One can ask if these values are representative for subjects who are exposed to vibration and heavy manual work. Therefore, it is recommended that QST is combined with a carefully conducted bedside physical examination [23].

The exposed workers had Work Ability Score (WAS) values exceeding 8 on a 10-point scale, both in 2018 and in 2022, indicating consistently good work ability. The greatest improvement of 1.2 units was seen in the unexposed group, but the increase was not statistically significant. Several studies have shown an association between work ability score and functional disability including reduced grip strength. The grip strength test is a useful tool to assess strength and functional capacity in healthcare workers [24].

Another important factor is age as WAS gradually weakens as you get older. We tried these two factors in the WAS-model, and they were both included. Other factors such as e.g. pain in hands and fingers, cramps in arms and hands and vibration exposure time were not included in the model.

It must, however, be remembered that a lot of other factors also can affect work ability. Some examples are high humidity and temperature, physical and work-related factors such as high workload, low job control and low social support [25, 26] and ergonomic factors as well as the worker’s social life.

The large decrease of musculoskeletal symptoms in neck-shoulder and hand-arm in unexposed workers can be an effect of ongoing work at the factory with the aim of reducing the static load and increasing the variation in work tasks, as well as an effect of the cessation of vibration exposure.

Forceful, repetitive work with non-neutral wrist postures increases the risk of e.g. carpal tunnel syndrome and epicondylitis [27]. Epidemiological evidence indicate that risk factors are interactive and often multiplicative. For musculoskeletal diseases in the upper extremity, the presence of two or more exposures multiplies the risk of an injury [27]. Epidemiological studies often have limited exposure variables. Deeper understanding of the underlying mechanisms and relationships can often be obtained if epidemiological studies are combined with controlled laboratory experiments.

The preventive measures initiated after the baseline examination in 2018 were well received at the factory. The enthusiasm was high in many areas, and large parts of the factory were involved in improvement of their working environment. It is also possible that the positive spirit among the workers during the joint preventive work may have influenced the WAS classification upwards.

### Limitations

The limited group sizes and the fairly short follow-up time were limiting factors as the healing process of vibration caused injuries may be quite long [6]. It was difficult to estimate the precise use of the modified tools on an individual basis, so this figure had to be estimated by using several different exposure estimation techniques, as earlier described. Neither was it possible to confirm the level of high frequency vibrations in every participant.

The strength of the study includes several items. The limited number of tools (impact wrenches and counter torque spanners) used at the factory is a positive factor. The effect of these tools is not disturbed by use of several other vibrating tools, which is common in industrial settings. The repeated work procedures at the factory and the use of component lists enable an accurate estimation of the actual exposure time. Another important factor is the low drop-out frequency of the workers which was around 8% during follow-up and the comprehensive examination and testing at the factory. There could also have been a positive effect from change of workstations and tempo.

There was as mentioned earlier great enthusiasm for the implemented preventive measures at the factory, which certainly facilitated the vibration isolation work. Another positive sign was that the number of newly diagnosed HAVS cases at the factory has decreased significantly in recent years.

## Conclusion

The vibration exposed workers showed no significant differences for 2-PD, monofilament, Jamar, and SWS classification during follow-up. However, a deterioration was observed for VPTs, needle tests and temperature sensitivity tests. Four years exposure to an A(8) value of 0.5–0.6 m/s^2^ means a continued low-grade vibration exposure, which can cause increased symptoms in sensitive individuals. The tests presented in Table 2 basically show no significant differences in the unexposed group when comparing the values ​​for 2018 and 2020, respectively. Due to the redesign of the impact wrenches and the counter torque spanners a reduction of the A(8)-values was noted, showing the efficiency of the relatively inexpensive preventive measures that was undertaken. During the follow-up period, the number of exposed workers who developed musculoskeletal disorders and newly reported cases of vibration injuries at the factory decreased.

These preventive measures made it possible to maintain the prevalence of vascular and neurosensory symptoms at a fairly constant level during the follow-up period. Without these preventive measures, a further progression of vascular and nerve symptoms at the factory most probably would have taken place.

## Data Availability

Availability of data and materialsThe dataset used and/or analysed during the current study are available from the corresponding author on reasonable request.
